# Tunability and Sensing Properties of Plasmonic/1D Photonic Crystal

**DOI:** 10.1038/srep41983

**Published:** 2017-02-08

**Authors:** Mohamed Shaban, Ashour M. Ahmed, Ehab Abdel-Rahman, Hany Hamdy

**Affiliations:** 1 Nanophotonics and Applications (NPA) Lab, Department of Physics, Faculty of Science, Beni -Suef University, Beni-Suef, 62514, Egypt; 2 Yousef Jameel Science and Technology Research Center, American University in Cairo, New Cairo, Cairo 11835, Egypt

## Abstract

Gold/one-dimensional photonic crystal (Au/1D-PC) is fabricated and applied for sensitive sensing of glucose and different chemical molecules of various refractive indices. The Au layer thickness is optimized to produce surface plasmon resonance (SPR) at the right edge of the photonic band gap (PBG). As the Au deposition time increased to 60 sec, the PBG width is increased from 46 to 86 nm in correlation with the behavior of the SPR. The selectivity of the optimized Au/1D-PC sensor is tested upon the increase of the environmental refractive index of the detected molecules. The resonance wavelength and the PBG edges increased linearly and the transmitted intensity increased nonlinearly as the environment refractive index increased. The SPR splits to two modes during the detection of chloroform molecules based on the localized capacitive coupling of Au particles. Also, this structure shows high sensitivity at different glucose concentrations. The PBG and SPR are shifted to longer wavelengths, and PBG width is decreased linearly with a rate of 16.04 Å/(μg/mm^3^) as the glucose concentration increased. The proposed structure merits; operation at room temperature, compact size, and easy fabrication; suggest that the proposed structure can be efficiently used for the biomedical and chemical application.

In past years, photonic crystals (PCs) are receiving increasing attention in both industry and academia due to their applications in photonics, electronics and biochemical. The PCs are a relatively new class of materials in which the refractive index is periodic in one-dimension (1D- PC), two-dimension (2D- PC), and three-dimension (3D-PC)^1^,^2^. The length scale of spatial periodicity is on the order of the wavelength of light propagating in the structure. When light waves incident on the PC, some photons are allowed to propagate through the PC and others are forbidden (totally reflected)^3^. The forbidden photon in particular frequency ranges leads to the appearance of stop bands in the transmission spectra. This usually referred to as photonic band gaps (PBGs), in analogy to the electronic band gaps of semiconductors^4^. Therefore, the PCs can be used to control light with fantastic facility and construct integrated optical devices that are impossible with conventional optics^5^. 1D-PCs are studied more attractive than 2D-PCs and 3D-PCs because of its simple fabrication and low cost^6^,^7^. For the 1D-PC synthesis, different methods such as plasma enhanced chemical vapor deposition (PECVD), radio frequency (RF) magnetron sputtering, spray pyrolysis deposition, thermal evaporation and some other has already been demonstrated. The PECVD offers several potential advantages over current processing technologies. Foremost is the opportunity to reduce deposition temperatures to below 400 °C^8^,^9^. Second, reactant species may be introduced into the low-pressure PECVD reactor by direct liquid injection. Third, it is more suitable for production high-quality thin films with excellent adhesion and controlling the layers thickness.

By monitoring PBG change or shift, photonic crystals have been used as optical sensors in many different areas such as environmental monitoring, chemical, and biological detections^10^. PCs sensors show high sensitivity, small size, and a wide dynamic range. Also, they are practical for on-chip integration with the incorporation of light sources and detectors^11^. Today, the refractive index (RI) is one of the most important optical parameters for the materials because it offers an indication of several physical and chemical changes. Hence, RI detection method is very important in industrially, environmentally, clinically, and to food processing monitoring^12^,^13^. In other side, quantitative detection of the glucose concentration (GC) has attracted much attention due to its significant index in clinical diagnoses, regulation of metabolism, and biochemical analysis^14^,^15^. For all of that, the development of simple, fast, cheap and high-performance sensors for the environment RI and GC has been the subject of concern for decades.

Since the first application of the surface plasmon resonance (SPR) phenomenon for sensing almost 30 years ago, this method has made significant advances in instrumentation development and applications^16^. Recently, SPR combined with PCs for active sensing of environmental RI change. Biswas *et al*. designed a new family of hollow-core photonic crystal fibers with embedded metal wires for sensitive refractive index measurement of fluids^17^. Otupiri *et al*. used numerical analysis to present a novel birefringent photonic crystal fiber biosensor constructed on the SPR model for sensing more than one analyte^18^. Zhao *et al*. reviewed the research developments of the photonic crystal fiber based surface plasmon resonance (PCF-SPR) chemical sensors^19^. Lately, Gao *et al*. presented a mode expansion method that can quantitatively describe guided resonances and bound states in the continuum (BICs) in periodic 1D-PC slabs, which is very promising for sensing applications^20^. From the previous attempts, photonic crystal fibers (such as the microstructured optical fiber, photonic bandgap fiber, and the Bragg fiber) with various structures were applied to design efficient SPR sensors.

However, very limited attempts have been made to combine 1D-PC with the SPR to improve the sensing properties. Baryshev and Merzlikin showed theoretically that a plasmonic 1D PC-based optical sensors exhibited good robustness and enhanced sensitivity in comparison with the conventional surface plasmon and Bloch surface wave sensors^21^. Lee *et al*. showed theoretically enhanced nonlinear optical effects due to the excitation of optical Tamm plasmon polaritons in 1D-PC structures^22^. Recently, Auguie *et al*. investigated with comprehensive numerical simulations the conditions for optimized coupling between light and Tamm plasmons excited at normal incidence from either side of the Au- distributed Bragg reflectors (DBR) structure^23^. So the optimized design of plasmonic 1D-PC opens up the possibility to increase the sensitivity of the PC^24^,^25^. In addition, a nanometer plasmonic layer such as Au layer will be serving as protection layer due to the Au has high resistance to oxidation and corrosion in various environmental conditions and hence improving the stability of the PC sensor^26^,^27^. Here, a simple 1D-PC terminated with Au nanostructured thin film is fabricated, characterized and used as an optical sensor for different chemical and biological molecules. Effect of the Au thickness and principles of optical sensing are discussed.

## Results and Discussion

### Characterization of the Au/1D-PC

**Figure 1:**
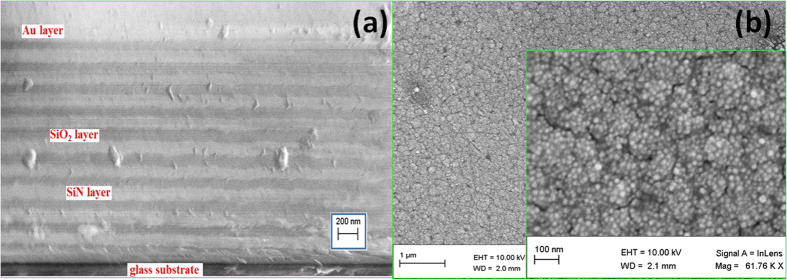
FE-SEM images of 1D-PC on a glass substrate coated with Au film for 60 sec (**a**) cross-sectional view and (**b**) top views at different magnifications.

Figure 1(a) shows a cross-sectional SEM image of a ten-layer 1D-PC made of SiO_2_/SiN deposited alternately on the glass substrate and terminated with Au layer. Multilayered stacks are clearly observed in this figure. The dark layers are the low refractive index layers (SiO_2_), and the white layers are the high refractive index layers (SiN). As seen in this figure, the thicknesses of SiO_2_, SiN, and Au layers are uniform and ~98, 112 and 126 nm, respectively.

Figure 1(b) shows top-view SEM images of the deposited Au layer at two different magnifications. As seen from these images, a uniform nanostructured Au layer covers the top surface of the 1D-PC. The PC surface is well covered with Au grains that are almost uniformly distributed over the surface. These grains are grown by the agglomeration of almost uniformly distributed spherical Au nanoparticles. These Au nanoparticles show a narrow size distribution and a very high density per unit area. The diameters of the nanoparticles are varied from 10.03 nm to 27.40 nm with an average of 18.34 nm.

**Figure 2:**
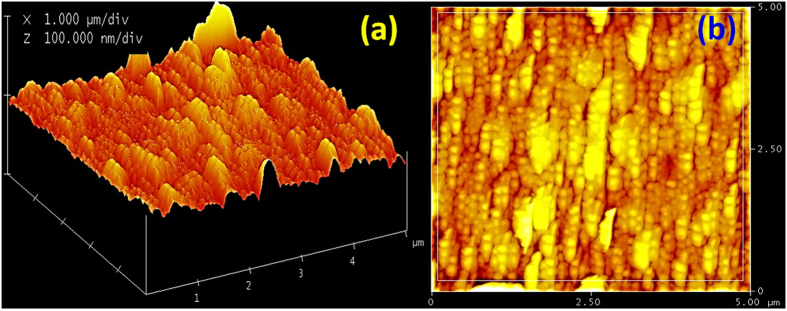
AFM images of the 1D-PC coated with Au film for 60* *sec.

The 3D and 2D AFM images of Au layer are shown in Fig. 2. The surface of the Au/1D-PC possesses peak and valley regions and are not absolutely flat surfaces. The bright areas show the overgrown Au crystallites of well-developed grain morphology. The Surface roughness is characterized by calculating the roughness parameters which are estimated by analyzing the topography scans of the sample’s surface. The roughness parameters include root mean square roughness (R_rms_ = 7.30 nm), average roughness (R_av_ = 5.58 nm), maximum peak height (R_p_ = 15.01 nm), maximum valley depth (R_v_ = 9.96 nm), and difference between R_p_ and R_v_ (R_p-V_ = 24.97 nm). Then, the proposed Au/1D-PC possesses good morphology and huge surface area with high density plasmonic hot spots. Then, the proposed structure may be used in different applications including sensors, catalysts, and optoelectronic components.

**Figure 3:**
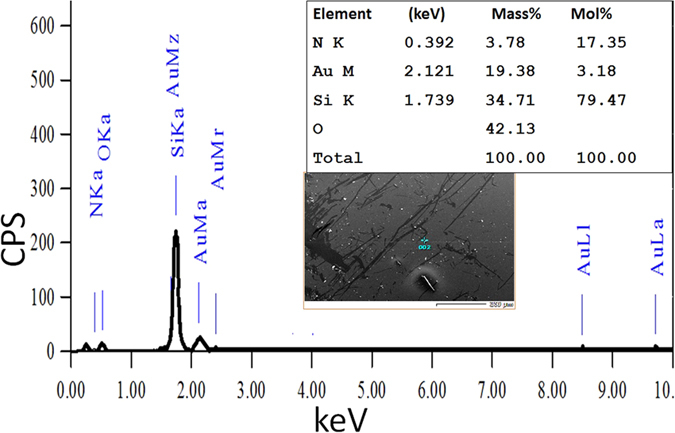
EDX spectrum of 1D-PC on a glass substrate coated with Au for 60 sec.

Qualitative and quantitative chemical composition of the fabricated 1D-PC coated with Au for 60 sec was studied using EDX. The EDX spectrum in Fig. 3 clearly confirms the presence of O, Si, Au, and N peaks. The quantitative results were 42.13% O, 34.71% Si, 19.38% Au, and 3.78% N, which indicates the high purity of the fabricated structures. No traces are detected from the glass substrate.

**Figure 4:**
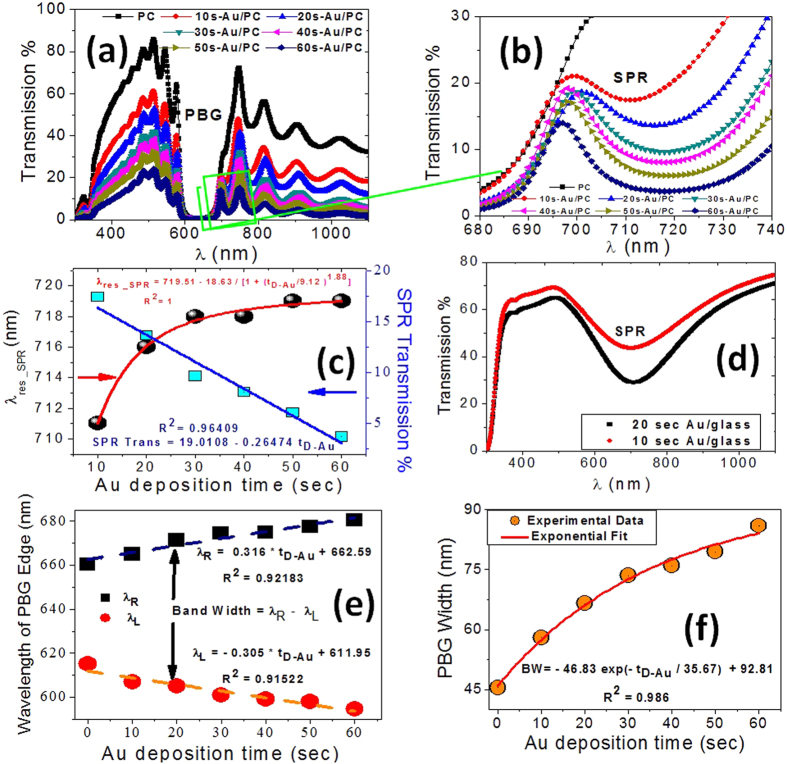
(**a**) Optical transmission of uncoated and Au-coated PC for sputtering time varied from 0 to 60 sec, (**b,c**) the wavelength position and transmission intensity of SPR mode versus the deposition time of Au, (**d**) transmission of a glass substrate coated with Au for 10 and 20 sec, (**e,f**) the left edge, right edge position and PBG width at various deposition time of Au.

From optical transmission of uncoated (SiO_2_/SiN)^10^ PC (black square and solid line) in Fig. 4(a), it can be seen that there is a PBG (i.e., a region of nearly zero transmittance). The left and right band edges of this PC are at λ_L_ = 615 nm and λ_R_ = 660 nm. The width of PBG is small, ∆λ_PBG_ = λ_R_ − λ_L_ = 45 nm because the PBG width is directly proportional to the refractive index contrast, Δn, between the two layers (SiN and SiO_2_)^28^. The formation of PBG can be explained by the interference of light, which is reflected by the periodic index variations. According to the optics theory, the light beam incident on the PC will be partly transmitted and partly reflected at each interface between SiN and SiO_2_. There are multiple reflections arise from the periodic variation of the refractive index of the PC. Then, the reflected waves produce multiple destructive or constructive interferences. The very high reflectivity (very low transmission) of light over a certain range of wavelengths is caused by constructive interference of the in-phase beams and called photonic band gap (PBG). Also, there are many ripples in the transmission response outside the PBG.

The optical response of the PC was observed to be markedly affected by the Au coating. There is a new minimum appear in the transmission spectrum of Au-PC around 723 nm at the right edge of the PBG as illustrated in Fig. 4(a and b). The same minimum was observed at 720 nm in the transmission spectrum of a glass slide coated with Au due to the SPR of Au layer, Fig. 4(d). The surface plasmon is a transverse-magnetic (TM) waves propagate at the metal-dielectric interface with electric field decaying exponentially in both media^29^. The surface plasmon wave (SPW) is excited when the free electrons in the conduction band of noble metal are induced to coherent oscillation by interacting electromagnetic fields of incident light^30^,^31^. At resonance, the energy of the incident photon is transferred for the excitation of the SPR, resulting in a sharp dip in the transmittance spectrum at a particular wavelength. The curved surface of the Au nanoparticle creates a sufficient restoring force on the conduction electrons^32^. Therefore, the SPR is characterized by substantial enhancements of the local electric fields surrounding the Au nanoparticles. The increasing in the local electric field intensity at the surrounding medium/Au and Au/PC interfaces will enhance the interaction between light and PC, and thus improves performance parameters of the PC as an optical sensor. The position of SPRs was shifted to longer wavelengths as the Au-deposition time increased, Fig. 4(c). This red shift may be ascribed to the size effect. As the size increased, the charge separation between the positive ion and the surface electron is increased. This leads to a decrease of the Coulombic restoring force which acting on the oscillating electrons. As a result, the energy of the plasmon resonance is reduced, and the peak of the plasmon resonance is red-shifted. Also, the transmittance of PC is decaying with increasing the thickness of Au film. This result from the dielectric constant of a noble metal such as Au is a complex with a relatively large imaginary part. The light absorption is increased as the thickness of Au layer increased. The absorption of noble metals is due to the electrons jumps between occupied bound d states and un-occupied hybridized sp states above the Fermi level in the visible range^33^.

With increasing Au thickness, the left band edge, λ_L_, is shifted to the shorter wavelength while the right band edges, λ_R_, is shifted to the longer wavelength and that causes an increase in the PBGs width as shown in Fig. 4(e and f). This is ascribed to the increase of the effective optical thickness of the structure after the Au deposition, which is the product of the thickness and the refractive index of the PC structure, according to the optical Bragg equation. According to the linear fitting of the experimental data, solid lines in Fig. 4(e), the shifts of λ_L_ and λ_R_ are given by the empirical equations;


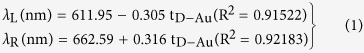


where t_D−Au_ the deposition time of Au in second and R^2^ is the coefficient of determination or the square of the correlation coefficient between the linear fitting and the experimental data at the right and left edges of the PBG. As shown the rate of shift of λ_R_ is greater than the rate of shift of λ_L_. This means that the existence of the SPR plays an important role in the tunability of the PBG width. Figure 4(f) clearly indicates a nonlinear increase of the PBG width and the best fitting for the experimental data is the exponential equation as shown in the figure.

### Refractive index sensing application

**Figure 5:**
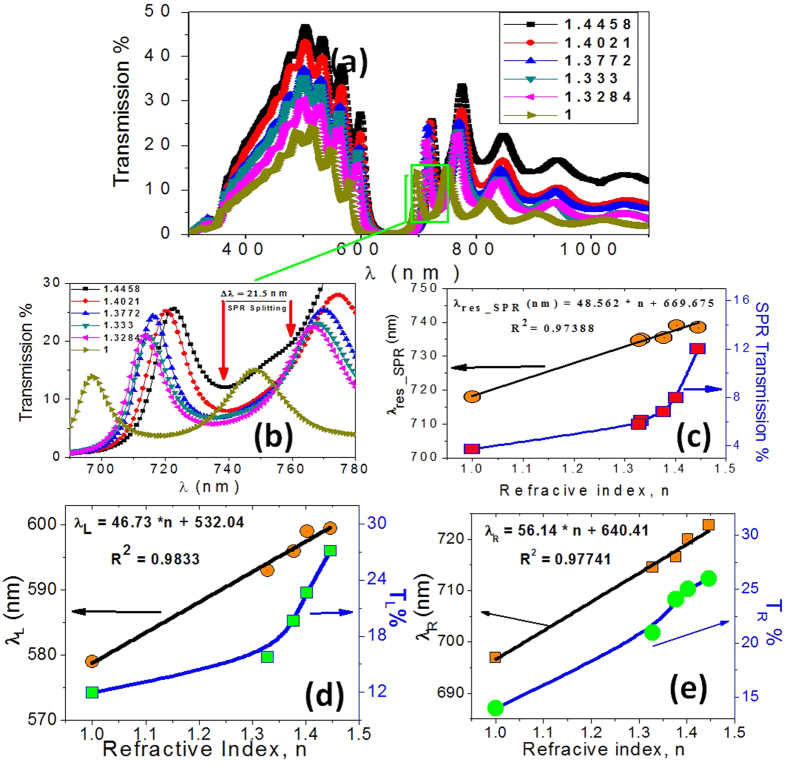
(**a**) Transmission spectra of Au/PC loaded with small amounts of liquids (<0.1 μl) of different refractive indices (**b,c**) the SPR wavelength and transmission intensity as function of RI of surrounding medium; variation of the position and transmission of the (**d**) left edge and (**e**) right edge of the PBG versus the environmental RI.

After fabrication and characterization of Au/PC, its performance as an optical sensor device for RI and GC was tested by immersing the PC into a series of solutions. Figure 5(a) shows the transmission spectra of Au/PC loaded with methanol, water, isopropanol, 2-methoxyethanol, and chloroform with RI of 1.3284, 1.333, 1.3772, 1.4021 and 1.4458, respectively. The PBG is red-shifted as the environmental RI increased. This ascribed to the decrease of the RI contrast as the environmental RI increased; hence both λ_R_ and λ_L_ are moving toward the longer wavelengths. Also, the transmittance of the PC increases with increasing the environmental RI. This is in agreement with previously reported results^34^. With increasing the environmental RI, the Fresnel reflection R, 
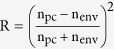
, at the PC/Au/environment interfaces decreases and hence the transmitted intensity increases as the environmental RI increased from 1.000 to 1.4458^35^,^36^.

Figure 5(b,c) shows the SPR wavelength and transmission intensity as a function of RI of surrounding medium. The SPRs are linearly shifted to longer wavelengths, and SPR transmission is exponentially increased as the refractive index of the environmental solution increased. This may be ascribed to two reasons. (1) The polarization of the adsorbed molecules of liquid due to the presence of electric field around the Au nanoparticle surface. This polarized medium compensates for some of the charges on the nanoparticle surface and can act as a bridge between the nanoparticles of Au layer to produce strong overlapping of Au local electric field. (2) The increasing of the refractive index for the environmental solution reduces the Coulombic restoring force that acting on the oscillation electrons in the Au particle. This reduction leads to a red shift in the plasmonic peak^37^. The linearity of the relationship between changes in guided resonance spectral location and surrounding RI indicates the symmetry of the fabricated sensor^38^. The black line in Fig. 5(c) is the linear fitting of the experimental data. The obtained empirical formula is


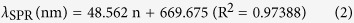


Also, there is a new minimum in the optical transmission is appear at 744.5 nm when the Au/PC loaded with chloroform of high RI (n = 1.4458) as indicated by rows in Fig. 5(b). The SPW strongly depends on the interaction of the molecules of sensing medium and Au nanoparticles. The chloroform has high adhesion so that when it comes in contact with the Au-PC surface, the molecules of chloroform get better adsorbed on Au surface. The hard binding of these molecules with the Au layer can enhance the inter-coupling between the Au nanoparticles. Then, the local electric field intensity around the Au particles is increased. Also, the presence of electric field around the Au nanoparticle surface also induces the polarization of the adsorbed chloroform molecules. A higher value of refractive index of chloroform results in more polarized charges for chloroform molecules. Hence, the polarized chloroform molecules can be acting as a bridge between the nanoparticles of Au layer. This produces strong overlapping of Au local electric field with the molecules of chloroform. Therefore, there is a redistribution of surface charges on adjacent Au nanoparticles which results in the splitting of SPR to two modes and the higher order SPR (new plasmon mode in the spectrum) can be achieved based on the localized capacitive coupling of Au particles. In this case, the separation between the SPR modes, Fig. 5(b), is Δλ = 21.5 nm.

Figure 5(d and e) show the variation of the position and transmission of left and right edge of PBG with the environmental RI. As the RI increased, the values of λ_L_ and λ_R_ are shifted toward longer wavelengths. The shifts can be linearly fitted according to the equations;


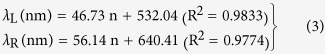


The performance of the PC as RI sensors is evaluated using the sensitivity parameter (S_n_) and signal-to-noise ratio (SNR). The sensitivity is given by


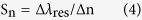


where Δλ_res_ is the resonance wavelength shift and Δn is the change in the environmental refractive index. The refractive index and resonance wavelength of air were used as references to calculate Δλ_res_ and Δn for the different liquid materials, i.e Δλ_res_ = λ_res_(liquid) − λ_res_(air) and Δn = n(liquid) − n(air).

Besides the sensitivity, signal-to-noise ratio (SNR), detection limit (δn), sensor resolution (SR) and figure of merits (FOM) are also important parameters to evaluate sensor performance. These parameters can be found using the following expressions^39^:


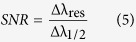



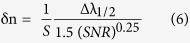



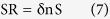



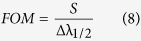


where Δλ_1/2_ is the full width at half maximum of the transmission dip.

Using Fig. 5 and Equations (4),(5),(6),(7),(8) S_n_, SNR, δn, SR, and FOM are calculated at the peak of surface plasmon resonance and shown in Table 1. From this Table, the average sensitivity is 50.23 nm/RIU. According to the linear fitting of the experimental data, the values of sensitivity, slope of the straight line, at λ_res_, λ_L_ and λ_R_ are 48.56, 46.73 and 56.14 nm/RIU, respectively. The observed trend is that longer wavelength guided resonances will have a higher sensitivity. This indicates 20% increase in the sensitivity of right PBG edge relative to that of left PBG. This may be attributed to the existence of the SPR very close to the right PBG edge which increases the spectral sensitivity and quality factors of right edge peak resulting in substantial enhancement of refractive index sensitivity. These sensitivities are better than that previously reported for photonic crystal fiber tip interferometer (11.5 nm/RIU over the RI range of 1.33–1.40)^12^, microfiber device (30.49 nm/RIU over the RI range of 1.334–1.348)^40^, Mach-Zehnder interferometer (15–40 nm/RIU)^41^,^42^,^43^, multilayer coated microsphere (maximum, 12.16 nm/RIU)^44^, and fiber grating sensor (below 5 nm/RIU)^45^. However, theoretical studies using finite different time domain (FDTD), COMSOL showed higher sensitivity than that shown here for more complicated proposed structures. These include photonic crystal waveguide-based sensors described by Bagci and Akaoglu (max Sn = 282.4 nm/RIU)^11^, sensors based on SPR in plastic optical fiber by Cennamo *et al*. (549 nm/RIU and 1325 nm/RIU)^46^, 2D photonic crystal slab biosensors by El-Beheiry *et al*. (max S_n_ = 902 nm/RIU for TM-like conditions)^38^, SPR-based optical fiber sensor by Al-Qazwini *et al*. (max Sn = 20310 nm/RIU)^47^, and multi-core flat fiber based surface plasmon resonance (MCFF SPR) by Rifat *et al*. (max Sn = 23000 nm/RIU)^48^.

### Detection of glucose concentration application

**Figure 6:**
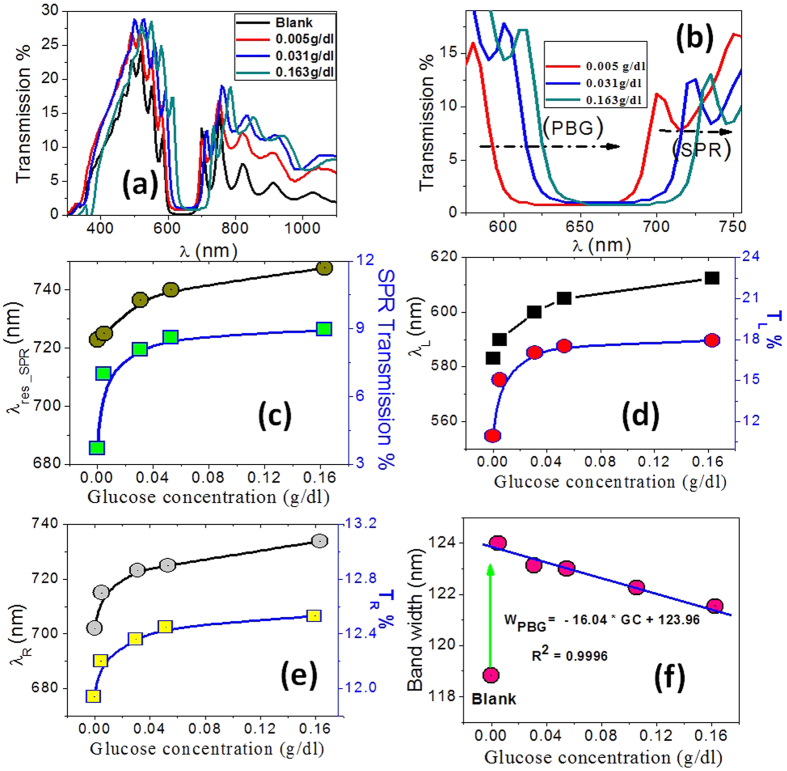
(**a,b**) Transmission spectra of Au-PC in response to the introduction of glucose solutions of different concentrations in the microfluidic channel (0.00, 0.005, 0.031 and 0.163 g/dl), (**c**) the guided wavelength and transmission intensity of SPR mode, (**d**) left edge, and (**e**) right edge of the PBG as function of GCs; and (**f**) variation of PBG width with the GCs.

The PC tested as an optical sensor device for glucose by immersing it into a series of aqueous GC solutions of different concentrations. Transmission spectra of PC immersed into glucose solutions with concentrations 0–0.163 g/dl are shown in Fig. 6(a and b). The spectral analyses of the transmission spectra are shown in Fig. 6(c–e) for the surface plasmon resonance (λ_SPR_), the left edge (λ_L_), and right edge (λ_R_) of the PBG. These figures clearly show a nonlinear shift of the guided wavelengths to longer wavelengths as the GCs increased. Also, the PBG is shifted toward higher wavelengths accompanied with an increase in the transmitted intensity at lower GCs and tiny increase or saturation at higher GCs as observed in Fig. 6. This behavior well correlates with the change of the RI contrast between the Au/PC and the environmental solution. Figure 6(c–e) depict the dependence of λ_SPR_, λ_L_ and λ_R_ and their corresponding transmitted intensities on GCs. For all guided wavelengths, the intensity of the transmission spectrum increased gradually while the surrounding GC was increased, but above 0.031 g/dl, the transmission is hardly changed. This may be assigned to the decrease of the reflected intensity as the concentration and, hence, the refractive index of the environment glucose layer increased.

The SGC, SNR, δn, SR, and FOM are calculated and shown in Table 2 at SPR for different environmental GCs. The refractive index and resonance wavelength of pure water were used as references to calculate Δλ_res_ and Δn for the different glucose concentrations. From this table, the average sensitivity is about 271 nm/g/dl and the maximum sensitivity is 407.42 nm/g/dl at a glucose concentration of 0.031 g/dl. The width of PBG is linearly decreased with the concentrations of glucose as shown in Fig. 6(f). The PBG width is reduced due to the movement speed of λ_R_ is faster than λ_L_ toward longer wavelengths with increasing the GC. This indicates the sensitivity of PC towards GC. The linear fitting of the experimental data in Fig. 6(f) yields the following linear relation


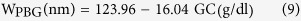


The slope of this curve is Δ W_PBG_/ΔCG = 16.04 nm/(g/dl), which give the sensitivity of PC for GC.

## Conclusion

Here, we have successfully designed an optical sensor based on 1D-PC terminated with plasmonic nanolayer by PECVD and sputter coating techniques. The fabricated sensor was efficiently applied for the detection of glucose of different concentrations and different chemical molecules of various refractive indices. The thickness of the Au nanolayer was successfully optimized to produce surface plasmon resonance (SPR) at the right edge of the photonic band gap (PBG) and to improve the sensitivity and stability of the proposed sensor. The morphology and chemical composition of the fabricated multilayered structure, Au/SiO_2_/SiN 1D-PC, was investigated. This sensor shows higher sensitivity ~50.23 nm/RIU and lower signal-to-noise ratio ~0.46 compared to previously reported experimental values for more complicated 1D-PC. 20% increase in the sensitivity of right PBG edge relative to that of left PBG edge was observed and ascribed to the existence of SPR very close to the right edge. The symmetry of the fabricated structure as RI sensor was confirmed by the linearity of the relationship between changes in guided resonance spectral location (λ_SPR_, λ_L,_ and λ_R_) and surrounding RI indicates. A new minimum in the optical transmission appears at 744.5 nm when the Au/PC loaded with chloroform of high RI (n = 1.4458) due to the splitting of SPR. Also, during the detection of glucose concentrations (GC), the PBG width decreased linearly with rate 16.04 Å/(μg/mm^3^ (and the guided spectral positions were red-shifted as the GC increased from 0 to 0.163 g/dl. The average sensitivity at SPR for GCs is about 271 nm/g/dl. These results indicate that the proposed plasmonic 1D-PC is suitable as an optical sensor for RI and GC. The significant advantages of the presented sensor; compact size, low fabrication cost, better performance regarding sensitivity and resolution, and large scale fabrication availability; suggest the use of this sensor for the biochemical and biomedical application.

## Methods

### Sensor fabrication

SiN layer was deposited using a PECVD system (PlasmaPro 100 Stratum, Oxford Instruments) with a gaseous mixture of high-purity (99.99%) SiH_4_/NH_3_/N_2_. The glass substrate has been heated to 200 °C during the deposition. To minimize the plasma damage of the film surface during the deposition, a high excitation frequency of 13.56 MHz was applied. 4.5% SiH_4_/95.5% N_2_ mixture was used as process gasses. The NH_3_ flow was fixed at 50 sccm, the pressure at 200 mTorr and the plasma power at 100 W during this experiment. For the second layer, SiO_2_ layer was formed by PECVD at 200 °C using SiH_4_/N_2_O gas at 100 W- RF power, making a film thickness of 100 nm using the same frequency.

Au layers of different thicknesses were deposited on the top surface of 1D-PC by DC sputtering at pressure 2 Torr and distance 8 cm in front of the Au target (99.99%) for different lengths of time (from 10 to 60 sec). The deposition rate of the Au layer on the 1D-PC is 2.1 nm/sec. For comparison, glass substrates were coated with the same Au layers, under the same conditions, to understand the effect of Au and its SPR on the optical properties of 1D-PC.

### Sensor Characterization

The morphological features of the 1D-PC were measured by using both field emission-scanning electron microscope (FE-SEM, ZEISS SUPRA 55 VP and ZEISS LEO, Gemini Column) and atomic force microscope (AFM, PARK SYSTEM, XE-100E). The chemical composition was studied using energy dispersive X-ray spectrometer (EDX; Oxford Link ISIS 300 EDX). Optical spectra in the spectral range from 300 to 1000 nm were measured with increment 1 nm using UV/VIS/NIR 3700 double beam Shimadzu spectrophotometer. All optical measurements in this research were conducted at the normal incident and room temperature.

### Sensing properties measurements

The sensing properties of the fabricated sensor were investigated by measuring transmission spectra of the Au/(SiN/SiO_2_)^10^/glass after loading the sample with small amounts of liquids (<0.1 μl) with different refractive indices. A schematic diagram of the experimental set-up (sample detection setup) is presented in the Supporting data; Fig. S1. The liquids were methanol, water, isopropanol, 2-methoxyethanol and chloroform with RI of the immersion liquid varying between 1.3284 and 1.4458. This RI range is very necessary for biomedical and chemical sensor application. Also, examine the optical response of the sensor to glucose solutions of different concentrations ranging from 0.009 to 0.163 g/dl. The glucose solution was prepared in DI water and sonicated to get a homogeneous mixture.

## Additional Information

**How to cite this article:** Shaban, M. *et al*. Tunability and Sensing Properties of Plasmonic/1D Photonic Crystal. *Sci. Rep.* **7**, 41983; doi: 10.1038/srep41983 (2017).

**Publisher's note:** Springer Nature remains neutral with regard to jurisdictional claims in published maps and institutional affiliations.

## Supplementary Material

Supplementary Information

## Figures and Tables

**Table 1:** Sensitivity parameter, S_n_, SNR, δn, SR, and FOM values for Au/1D PC sensor obtained for different environmental refractive indices.

n_s_	Δn_s_ (RIU)	λ_res_ (nm)	Δ λ_res_ (nm)	Δ λ_1/2_ (nm)	SNR = Δ λ_res_/Δ λ_1/2_	S_λn_ = Δλ_res_/Δn (nm/RIU)	δn	FOM	SR
1	—	718	—	36	—	—			
1.3284	0.3284	734.5	16.5	37.5	0.44	50.24	0.610	1.339	30.646
1.333	0.333	735	17	37.75	0.45	51.005	0.602	1.351	30.705
1.3772	0.3772	735.5	17.5	38.4	0.46	46.394	0.670	1.208	31.084
1.4021	0.4021	739.5	21.5	35.6	0.60	53.469	0.504	1.501	26.948

**Table 2:** Sensitivity parameter, S_GC_, SNR, δn, SR, and FOM values for Au/1D PC sensor obtained for different environmental glucose concentrations.

GC (g/dl)	∆ GC (g/dl)	λ_res_ (nm)	Δ λ_res_ (nm)	Δλ_1/2_ (nm)	SNR = Δλ_res_/Δ λ_1/2_	S_GC_ = Δλ_res_/ΔGC (nm/g/dl)	δn	FOM	SR
0		723.86		21.32					
0.005	0.005	725	1.14	18.89	0.06	228	0.1116	12.0966	25.4448
0.031	0.031	736.49	12.63	24.79	0.50	407.42	0.0482	16.4349	19.6376
0.053	0.053	740	16.14	26	0.62	304.52	0.0641	11.7123	19.5197
0.163	0.163	747.55	23.69	34.3	0.69	145.33	0.1726	4.2370	25.084
