# Supplementary material for: Tunability and Sensing Properties of Plasmonic/1D Photonic Crystal
Source: Sci Rep. 2017 Feb 8;7:41983. doi: 10.1038/srep41983 (PMC5296759; doi:10.1038/srep41983)
Supplement: Supplementary Information [file srep41983-s1.doc]

**Supplementary Data**

**Tunability and Sensing Properties of Plasmonic/**

**1D Photonic Crystal**

Mohamed Shaban^1,*^, Ashour M. Ahmed^1^, Ehab Abdel-Rahman^2^, Hany Hamdy^1^

^1^ Nanophotonics and Applications (NPA) Lab, Department of Physics, Faculty of Science, Beni -Suef University, Beni-Suef, 62514, Egypt

^2^ Yousef Jameel Science and Technology Research Center, American University in Cairo, New Cairo, Cairo 11835, Egypt


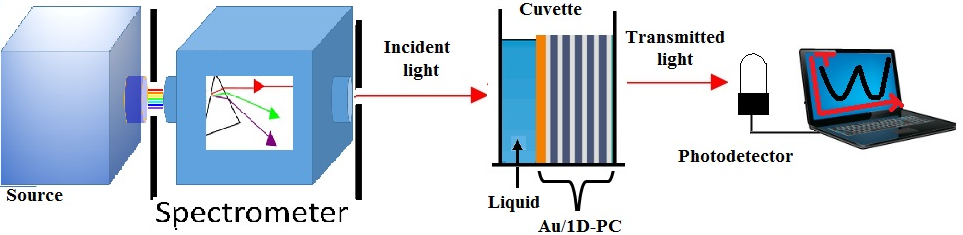


Figure S1 **A schematic diagram of the experimental sensing set-up (sample detection setup).**
